# Association between Impulsivity and Weight Status in a General Population

**DOI:** 10.3390/nu9030217

**Published:** 2017-03-01

**Authors:** Marc Bénard, Géraldine M. Camilleri, Fabrice Etilé, Caroline Méjean, France Bellisle, Gérard Reach, Serge Hercberg, Sandrine Péneau

**Affiliations:** 1 Equipe de Recherche en Epidémiologie Nutritionnelle, Centre de Recherche en Epidémiologie et Statistiques, Université Paris 13, INSERM U1153, INRA U1125, Cnam, COMUE Sorbonne Paris Cité, Bobigny 93017, France; g.camilleri@eren.smbh.univ-paris13.fr (G.M.C.); c.mejean@eren.smbh.univ-paris13.fr (C.M.); f.bellisle@eren.smbh.univ-paris13.fr (F.B.); s.hercberg@eren.smbh.univ-paris13.fr (S.H.); 2 Paris School of Economics and INRA, UMR1393 PjSE, 48 Boulevard Jourdan, Paris 75014, France; fabrice.etile@inra.fr; 3 INRA MOISA UMR1110, Montpellier 34000, France; 4 Service d’Endocrinologie, Diabétologie, Maladies Métaboliques, Hôpital Avicenne, Bobigny 93017, France; gerard.reach@aphp.fr; 5 Unité de Surveillance et d’Epidémiologie Nutritionnelle, Institut de Veille Sanitaire, Université Paris 13, Bobigny 93017, France; 6 Département de Santé Publique, Hôpital Avicenne, Bobigny 93017, France

**Keywords:** impulsivity, overweight, gender

## Abstract

The objective of this study is to examine the association between impulsivity and weight status in a large sample of the adult general population in France, and the influence of gender on this relationship. A total of 11,929 men and 39,114 women participating in the NutriNet-Santé cohort were selected in this cross-sectional analysis. The Barratt Impulsiveness Scale (BIS-11) was used to assess impulsivity. Weight and height were self-reported. The association between impulsivity and BMI was estimated using logistic regressions adjusted for socio-demographic and lifestyle factors. Individuals with high impulsivity levels (BIS-11 total score >71) were more likely to be obese (Odds Ratio (OR) = 1.80, 95% Confidence Interval (CI): 1.39, 2.33 in men; OR = 1.30, 95% CI: 1.15, 1.48 in women) compared to individuals in the average range of impulsivity. The strongest associations between impulsivity and obesity were observed in men, where highly impulsive participants were more likely to be class III obese (BMI > 40 kg/m^2^) (OR = 3.57, 95% CI: 1.86, 6.85). This large sample analysis supports the existence of a relationship between impulsivity and weight status and the importance of psychological factors in the prevention of obesity.

## 1. Introduction

Overweight and obesity currently represent a major global health burden and are leading public health issues in many countries [1], with associated comorbidities such as cardiovascular disease, type 2 diabetes and cancers [2]. Numerous influences contribute to the development of overweight and obesity, among which psychological and behavioral factors.

Impulsivity is a personality trait that is defined as “a predisposition toward rapid, unplanned reactions to internal or external stimuli without regard to the negative consequences of these reactions to the impulsive individuals or to others” [3]. It is a multidimensional construct measured with either self-report questionnaires or behavioral tasks [3,4,5]. The Barratt Impulsiveness Scale Version 11 (BIS-11) [6] is the most often used self-report questionnaire to assess impulsivity.

According to this definition, impulsive individuals could present a tendency to have rapid and unplanned reactions toward food, which could lead to overeating and thus obesity. Findings on the association between impulsivity, as measured by the BIS-11, and weight status, however, are limited and divergent. A study has shown a significant higher impulsivity level among overweight subjects compared to normal weight subjects [7]. However, other studies found no significant difference in impulsivity between obese and normal weight participants [8], and no significant correlation between impulsivity and BMI [9,10]. It must be noted that these previous studies were performed on small and specific samples. Divergences in the characteristics of these samples, such as eating disorders [11] or socio-demographic and lifestyle characteristics, could explain these differences. Studies assessing impulsivity using self-report questionnaires [12,13,14,15,16,17] other than the BIS-11, or behavioral tasks [18,19] have shown a link with weight status. Impulsive personality has also been associated with a tendency to overeat [4,20,21,22] and with eating disorders [23,24,25].

The potential effect of gender on the association between impulsivity and weight status has rarely been investigated, although gender is known to modify the association between personality dimensions and weight status [26].

The aim of this study was to explore the association between impulsivity using the BIS-11 and weight status, in a large sample of the general French population participating in the NutriNet-Santé cohort. Furthermore, we also assessed whether gender could modify this association.

## 2. Materials and Methods

### 2.1. Population

This study was conducted as part of the NutriNet-Santé study, which is a large ongoing web-based prospective cohort started in France in May 2009 with approx. 160,000 participants. The rationale, design and methods of the study have been described elsewhere [27]. Its aim is to explore the relationships between nutrition and health, and more precisely cardiovascular diseases, cancers, mortality and the determinants of eating behavior and nutritional status. Participants are adult volunteers (age ≥18 years) of the general French population with a follow-up of at least 10 years. At inclusion, participants have to complete several self-reported web-based questionnaires to assess their diet, their physical activity, anthropometric measures, lifestyle characteristics, socioeconomic conditions and health status. Participants complete this set of questionnaires every year after inclusion. Finally, another set of optional questionnaires related to determinants of eating behaviors and nutritional status are sent to every participant each month. This study was conducted in accordance with the Declaration of Helsinki, and all procedures were approved by the International Research Board of the French Institute for Health and Medical Research (IRB Inserm n° 0000388FWA00005831) and the Commission Nationale Informatique et Libertés (CNIL n° 908450 and n° 909216). Electronic informed consent was obtained from all participants.

### 2.2. Measures

#### 2.2.1. Impulsivity

To assess impulsivity, the French version of the BIS-11 questionnaire (derived from the French version of the BIS-10 questionnaire) [28] was administered to all participants of the NutriNet-Santé study in June 2014 with a maximum answer delay of 6 months. The BIS-11 is the most often used self-report questionnaire to assess impulsivity [29]. It is a 30-item self-report questionnaire [6] developed to assess the personality construct of impulsivity and its 3 aspects: attentional impulsiveness (focusing on the task at hand), motor impulsiveness (acting without thinking) and non-planning impulsiveness (lack of orientation to the future). Each item is measured on a 4-point Likert scale ranging from “rarely/never” to “almost always/always”. The total score is obtained by summing all the item ratings, offering a possible range from 30 to 120, with higher scores indicating greater impulsivity. In our population, the BIS-11 displayed good internal consistency (Cronbach’s α = 0.78). However, the three subscales had relatively low reliability with Cronbach’s α coefficients of 0.58 for the attentional subscale, 0.57 for the motor subscale, and 0.64 for the non-planning subscale. Moreover, confirmatory factor analysis revealed a weak 3-dimension structure of the BIS-11 in our data (Standardized Root-Mean square Residual (SRMR) = 0.07, Adjusted Goodness-of-Fit Index (AGFI) = 0.88, Normed-Fit Index (NFI) = 0.70). Therefore, we only considered the total BIS-11 score in the present study. We divided this score into 3 different categories according to the clinical thresholds proposed by Stanford et al. [29]. Subjects with a BIS-11 total score >71 were considered highly impulsive, those between 52 and 71 were in the average range of impulsivity, and those <52 were defined as over-controlled. 

#### 2.2.2. Anthropometry

Self-reported height and weight data were collected each year using a web-based questionnaire. Closest available data after the date of completion of the BIS-11 were used. BMI (kg/m^2^) was calculated as the ratio of weight to squared height. According to WHO reference values [1], participants were classified into four different weight statuses following their BMI: underweight (BMI < 18.5), normal (18.5 ≤ BMI < 25), overweight (25 ≤ BMI < 30), and obese (BMI ≥ 30). Obese individuals were further classified into three obese categories: obese class I (30 ≤ BMI < 35), obese class II (35 ≤ BMI < 40) and obese class III (BMI ≥ 40).

#### 2.2.3. Covariates

Potential confounders of the relationship between impulsivity and weight status identified based on the literature were considered: age (18–24 years, 25–39 years, 40–54 years, 55–64 years, ≥65 years) [30], education level (primary, secondary, university) [30], smoking status (never smoker, former smoker or current smoker) [31], alcohol intake (g/day) [31], physical activity [32], occupational status (unemployed, student, self-employed and farmer, employee and manual worker, managerial staff and intellectual profession, intermediate professions, retired) [30], monthly income per household unit [30], matrimonial status (living alone, living with a partner, married, divorced or separated or widowed [30]. Physical activity was assessed with a short form of the French version of the International Physical Activity Questionnaire (IPAQ) [33]. Weekly energy expenditure expressed in Metabolic Equivalent of Task (MET-minutes/week) was estimated and three levels of physical activity were constituted (low (<30 min/day), moderate (30–60 min/day), and high (≥60 min/day)). Monthly income per household unit was calculated with information about household income and composition. The number of people of the household was converted into a number of consumption units (CU) according to a weighting system: one CU is attributed for the first adult in the household, 0.5 for other persons aged 14 or older and 0.3 for children under 14 [34]. Categories of income were defined as followed: <900, 900–1200, 1200–1800, 1800–2300, 2300–2700, 2700–3700, and >3700 euros per household unit as well as “unwilling to answer”.

### 2.3. Statistical Analyses

Subjects’ characteristics were compared across gender with Student’s t-tests for continuous variables and chi-square tests for categorical variables. Since the test for the proportional odds assumption was significant (*p* < 0.0001), multinomial logistic regression analyses were performed to assess the association between impulsivity categories and BMI categories (as a 4-level dependent variable with normal weight category as reference). An additional analysis was performed on the three obese categories. The strength of the association was determined by calculating odds ratios (ORs) and 95% confidence intervals (CI). An interaction between impulsivity and gender was examined in the multinomial logistic model. Since this interaction was significant (*p* < 0.0001), all models were stratified by gender. Two models were tested: a model without adjustment and a model adjusted on covariates. The adjusted model included the following variables: age, education level, smoking status, alcohol intake, physical activity, employment status, monthly income per household unit and matrimonial status. Missing data on covariates were imputed using multiple imputation. The Hochberg method was applied to correct for multiple testing. All tests of statistical significance were 2-sided and significance was set at 5%. Statistical analyses were performed using SAS software (version 9.3, SAS Institute Inc., Cary, NC, USA). 

## 3. Results

### 3.1. Characteristics of the Sample

A total of 51,394 subjects (41%) from the NutriNet-Santé cohort completed the optional BIS-11 questionnaire, from the 125,377 subjects who received it. Among them, 73 participants were excluded because of missing data on weight or height, 15 participants because they presented an acquiescence bias (agreeing to all questions without consideration of reversed items) in answers of the BIS-11, and 263 because they were pregnant, leaving 51,043 participants (11,929 men and 39,114 women) in the final data set. Compared with excluded participants, included participants were older (50.5 years for included participants vs. 42.8 years for excluded participants, *p* < 0.0001), had a higher proportion of men (23.4% vs. 21.8%, *p* < 0.0001) and a higher proportion of individuals with university education (64.2% vs. 58.8%, *p* < 0.0001). The proportion of overweight participants (excluding obesity) was higher (23.2 vs. 21.4%, *p* < 0.0001), while the proportion of obese was lower (9.4% vs. 11.5%, *p* < 0.0001). The percentage of underweight was equal across samples (5.0% vs. 5.2%, *p* = 0.068).

Table 1 shows characteristics of the sample according to gender. Overall, women were younger, had better level of education, more often had never been a smoker, drank less alcohol, had a better level of physical activity, were more active, had lower levels of income in their household, were less often married, and were less overweight compared to men. Women were also found to be significantly more impulsive than men on average, even when adjusting for potential confounders (57.5 for men vs. 59.1 for women).

### 3.2. Socio-Demographic, Economic and Lifestyle Factors According to the Level of Impulsivity

Table 2 shows individual characteristics according to the BIS-11 total score and the BIS-11 categories. Mean of the BIS-11 total score for the whole sample was 58.7 (SD = 8.0, range = 31–102). Highly impulsive subjects represented 6.3% of the sample, whereas over-controlled subjects represented 17.8% of the sample. The percentage of highly impulsive subjects was high in younger participants, in women, in participants with a lower level of education, in current smokers, in participants with a low level of physical activity, in unemployed subjects or student, in participants with a low monthly income per household unit and in participants living alone. Moreover, highly impulsive respondents had the highest consumption of alcohol. The percentage of highly impulsive subjects increased with level of overweight/obesity and was highest in class III obesity. Percentage of over-controlled subjects was higher in underweight and normal-weight subjects than in overweight/obese respondents.

### 3.3. Association between Impulsivity and BMI Categories According to Gender

We observed a significant interaction between gender and impulsivity (*p* < 0.0001). Results of the positive association between impulsivity and BMI stratified by gender are shown in Table 3. In both genders, individuals with high impulsivity level (total BIS-11 score >71) were more likely to be obese. Impulsive men were also more likely to be overweight. The highest OR were observed for class III obesity. For highly impulsive women, associations between impulsivity and the 3 classes of obesity were non-significant. Associations between over-control and weight status were all non-significant.

## 4. Discussion

This population-based study showed that impulsive men were more likely to be overweight and obese and impulsive women were more likely to be obese compared to participants with an average level of impulsivity. These associations were particularly strong for obesity and more specifically for class III obesity in men. Women were more impulsive than men, but the association between impulsivity and weight status was stronger in men.

### 4.1. Level of Impulsivity According to Socio-Demographic, Economic and Lifestyle Characteristics

The average score of the BIS-11 in our study was consistent with previous studies [29,35]. In agreement with the literature, higher levels of impulsivity were observed in younger participants, [30] low-educated subjects [30], smokers [31], higher alcohol drinkers [31], unemployed subjects and students [30], participants with low income [30] and in participants living alone [30]. Higher levels of impulsivity were also found in subjects with lower physical activity, but the difference was small. Finally, women had significantly greater scores of impulsivity than men, contrasting with previous data showing no differences in impulsivity measured by the BIS-11 according to gender [6,9,29,36]. A meta-analysis [37] found slightly higher levels of impulsivity in men using the BIS-11. However, differences in impulsivity according to gender varied depending on the measure of impulsivity or the factor that was studied.

### 4.2. Association between Impulsivity Levels and Weight Status

In agreement with our study, impulsivity measured by the BIS-11 was lower in a healthy weight group compared with an overweight group without taking into account potential confounders [7]. However, another study found no difference in impulsivity between obese and normal weight subjects [8]. Other studies reporting correlation coefficients between the BIS-11 (or a short version of the BIS-11) and BMI showed either non-statistically significant correlations [9,10] or weak correlations [16,38,39]. It must be noted that these studies [9,10,16,38,39] were performed on specific populations (generally women and/or students) with a lack of heterogeneity which could alter the association. Studies using other measures of impulsivity, including self-reported personality trait questionnaires [12,13,14,15,17], and behavioral measures, such as response inhibition [18] or delay discounting [19,40], found positive associations between impulsivity and weight status.

Impulsivity could indicate a lack of consideration about health consequences when choosing food. Impulsive individuals with a high reward sensitivity will rather favor present benefit and consume foods that give them pleasure [41]. In the literature, impulsive behavior was linked to overeating and preference for sweet and fatty foods [42,43,44], which could lead to weight gain over time. Impulsivity was related to other eating-behavior facets, for instance emotional eating and external eating [45,46], and mood before eating [47]. Impulsive individuals also tend to exhibit pathological eating behaviors such as binge eating [48], addictive-like consumption of food [49], and other eating disorders [24,25] more often than non-impulsive ones. Impulsive eaters with high dietary restraint were shown to overeat more than non-impulsive high-restrained eaters [22], while another study found no interaction between dietary restraint and impulsivity [50]. Food reward responsivity (FFR) predicted a higher BMI among impulsive individuals compared with non-impulsive individuals [50]. Finally, specific interactions with reward sensitivity, inhibitory control and emotion regulation interactions might also occur [9,41]. These data suggest that impulsive behavior could therefore lead to difficulties in control of food intake and weight management.

In the present study, impulsivity was shown to be strongly associated with severe obesity in men, whereas the association was moderate with overweight. Interestingly, a high prevalence of attention deficit hyperactivity disorder (ADHD)—a disorder characterized by impulsive behavior [38]—was reported among people of extreme obesity [51,52]. ADHD has good correlation with the BIS-11 and is often linked to overeating and overweight [53].

We cannot exclude potential reverse or bi-directional associations. Indeed, weight gain has also been associated to a change in personality and more specifically an increase in impulsivity [54], which could be another explanation for the higher proportion of severely obese men among highly impulsive ones compared with other weight status.

We found no association between an over-controlled personality and weight status. This result differed from a previous study, which found a protective impact of a high level of “conscientiousness” on weight gain [15]. Gerlach defined conscientiousness as a “measure of regulation of internal urges and self-discipline”, which may be a “potential source of control over impulsive reward-oriented behavior” [15]. Another study found a similar result with impulsivity measured with the Revised NEO Personality Inventory, where low impulsiveness was a predictor of underweight three years after the personality assessment [17]. These findings suggest that an over-controlled behavior may lead to over-controlled food consumption, leading, in some cases, to underweight. Even though the associations between high impulsivity and underweight were non-significant in our study, results tended to indicate higher odds of being underweight for impulsive participants. 

### 4.3. Gender Differences

Men were more likely to be overweight or obese than women when they were highly impulsive. To our knowledge, only one study assessed the association between the BIS-11 and weight status stratified by gender. It found an association with BMI in women whereas no association was found in men [55]. Gender was shown to modify the association between psychoticism and extraversion personality dimensions, which are closely linked to impulsivity and weight status [26]. Our data showed stronger associations for severe obesity in both genders, even though these associations were non-significant for women after correction for multiple testing, suggesting a weaker and maybe different relationship with impulsivity. Women generally have a higher concern for their body shape and weight control compared to men, which could inhibit impulsive behaviors towards food. Finally, gender was shown to modify the association between weight status and eating behaviors related to impulsivity such as disinhibition [56], external eating [57] and emotional eating [57,58]. Higher cognitive dietary restraint in women [56] could also explain the weaker relationship between impulsivity and weight status in women. These results emphasize the importance of considering gender when assessing the association between personality traits or behavior and weight status and in prevention strategies.

### 4.4. Strengths and Limitations

The main strength of this study is its large sample size with subjects of various socio-demographic characteristics and nutritional status, which allows the use of multiple covariates to adjust for confounding factors and confers a great statistical power to detect significant differences among several classes of weight status. This study brings information about the importance of socio-demographic, economic and lifestyle confounding factors, which have rarely been studied when evaluating the association between impulsivity and nutritional status. However, we cannot rule out the possibility that other important confounders, such as individual or environmental factors, were not taken into account. To our knowledge, few studies assessed the association between impulsivity and weight status in men and women separately. One limitation of our study is its cross-sectional design, which does not allow us to assess causality. Another limit is the self-reported anthropometric measures, which could have led to misclassification. However, standardized clinical measurements on a subsample (*n* = 2513) of the NutriNet-Santé cohort showed a good convergence with self-reported data [59]. Our study could also present a selection bias because of the method used to recruit participants, which was based on volunteering. Consequently, our subjects may have high health awareness compared to the global population, and may not be representative of the French population. Considering the multidimensionality of impulsivity as a construct and its various methods of measurement, caution is needed when interpreting and extrapolating our results. The BIS-11 questionnaire is one of the most used self-report measures of impulsivity even though its three subtraits have rarely been explored [29]. In our sample, two out of the three BIS-11 subscales showed insufficient internal consistency, suggesting a low reliability of the questionnaire in our sample in the assessment of impulsivity. However, several studies showed similar issues [35,60]. Finally, the presence of subjects with psychiatric (e.g., bipolar) or psychological disorders could introduce a bias in our analysis since they have not been excluded [29].

## 5. Conclusions

These results showed an association between high levels of impulsivity measured by the BIS-11 and weight status, where highly impulsive participants were more likely to be overweight or obese. The strongest associations were found in men and with severe obesity. These findings underline disparities regarding these associations according to gender and weight status, suggesting that the mechanisms of the relationship between weight status and impulsivity could be different for men and women. Future studies should investigate gender differences and take them into account when assessing the relationship between impulsivity and weight status. Our results support the need to consider individual personality traits and characteristics in overweight/obesity prevention and treatment. Strategies such as inhibitory control training or attentional bias modification protocols could represent a way to reduce impulsive behaviors toward food.

## Figures and Tables

**Table 1:** Individual characteristics of 51,043 participants of the NutriNet-Santé study (2014), according to gender.

Variables	All (*n* = 51,043)	Men (*n* = 11,929)	Women (*n* = 39,144)	*p* ^1^
Age (years) ^2^	50.5 (14.6)	55.9 (14.1)	48.8 (14.3)	<0.0001
Age categories (%)				<0.0001
(18–24)	3.2	1.5	3.8	
(25–39)	23.3	14.8	25.8	
(40–54)	28.8	19.7	30.2	
(55–64)	24.8	23.3	24.8	
65+	19.9	40.6	15.4	
Education level (%)				<0.0001
Primary	2.5	3.1	2.3	
Secondary	30.1	32.4	29.4	
University	67.2	64.4	68.1	
Missing data	0.2	0.1	0.2	
Smoking status (%)				<0.0001
Never smoker	50.8	41.5	53.6	
Former smoker	36.0	46.5	32.8	
Current smoker	13.2	12.0	13.5	
Missing data	<0.1	0.0	<0.1	
Alcohol intake (g/day) ^2^	7.0 (9.9)	12.6 (14.0)	5.3 (7.4)	<0.0001
Physical activity (%)				<0.0001
Low	35.5	44.7	32.6	
Moderate	41.7	35.8	43.4	
High	22.7	19.3	23.8	
Missing data	0.2	0.1	0.2	
Occupational status (%)				<0.0001
Unemployed	9.6	4.4	11.2	
Student	2.8	1.3	3.2	
Self-employed, farmer	2.0	2.3	1.9	
Employee, manual worker	15.4	8.2	17.7	
Managerial staff, intellectual profession	23.5	26.3	22.7	
Intermediate professions	15.9	10.7	17.5	
Retired	30.8	46.9	25.9	
Missing data	<0.1	<0.1	<0.1	
Monthly income per household unit (%) ^3^				<0.0001
Less than 900 €	7.1	4.7	7.8	
Between 900 € and 1200 €	4.6	3.4	4.9	
Between 1200 € and 1800 €	21.4	19.7	22.0	
Between 1800 € and 2300 €	15.0	14.1	15.3	
Between 2300 € and 2700 €	10.0	11.3	9.6	
Between 2700 € and 3700 €	16.9	20.3	15.8	
More than 3700 €	12.2	17.4	10.6	
Unwilling to answer	12.2	8.8	13.2	
Missing data	0.7	0.3	0.8	
Matrimonial status (%)				<0.0001
Living alone	13.9	10.7	14.9	
Living with a partner	20.6	16.8	21.7	
Married	53.5	63.9	50.3	
Divorced, separated, widowed	12.0	8.4	13.1	
Missing data	0.1	0.2	0.1	
BMI (kg/m^2^) ^2^	24.0 (4.5)	25.1 (3.8)	23.6 (4.7)	<0.0001
Nutritional status (%)				<0.0001 ^4^
Underweight (<18.5 kg/m^2^)	5.0	1.0	6.2	
Normal (18.5–24.99 kg/m^2^)	62.4	54.2	64.9	
Overweight (25–29.99 kg/m^2^)	23.2	35.1	19.6	
Obese (≥30 kg/m^2^)	9.4	9.7	9.3	
Obese Class I (30–34.99 kg/m^2^)	6.5	7.5	6.2	
Obese Class II (35–39.99 kg/m^2^)	2.0	1.6	2.2	
Obese Class III (≥40 kg/m^2^)	0.9	0.6	0.9	
BIS-11 total score ^2^	58.7 (8.0)	57.6 (8.0)	59.1 (8.0)	<0.0001

Boldface indicates statistical significance; ^1^ Adjusted p values for multiple comparisons (Hochberg method) based on the Student’s t test and Pearson χ^2^ test, as appropriate; ^2^ Mean (SD); ^3^ The household income per month was calculated by consumption units (CU). The number of people of the household was converted into a number of CU according to a weighting system: one CU is attributed for the first adult in the household, 0.5 for other persons aged 14 or older and 0.3 for children under 14 [34]; ^4^ The test was performed for underweight, normal, overweight and global obesity categories.

**Table 2:** Individual characteristics of 51,043 participants of the NutriNet-Santé study (2014), according to the BIS-11 total score and its categories, according to gender.

Variables	Whole Sample	*p* ^1^	Men (*n* = 11,929)			*p* ^1^	Women (*n* = 39,144)			*p* ^1^
	BIS-11 Mean (SD)		Over-Control (*n* = 2641)	Average Impulsivity (*n* = 8691)	High Impulsivity (*n* = 597)		Over-Control (*n* = 6460)	Average Impulsivity (*n* = 30,051)	High Impulsivity (*n* = 2603)	
Age (years) ^2^			55.8 (13.5)	55.9 (14.3)	55.8 (14.6)	0.959	49.0 (13.8)	48.9 (14.3)	48.3 (15.2)	0.18
Age categories (%)		<0.0001				0.02				<0.0001
(18–24)	60.8 (8.4)		16.6	74.3	9.1		11.4	78.1	10.6	
(25–39)	58.8 (7.9)		20.6	74.9	4.5		17.0	76.5	6.6	
(40–54)	58.5 (8.0)		23.9	71.1	5.0		17.1	76.6	6.2	
(55–64)	58.7 (7.9)		23.1	72.2	4.7		16.3	77.4	6.4	
65+	58.8 (8.2)		21.2	73.6	5.3		16.3	76.6	7.1	
Education (%)		<0.0001				<0.0001				<0.0001
Primary	61.5 (8.7)		17.3	70.5	12.2		8.8	77.0	14.1	
Secondary	59.8 (8.3)		19.1	74.2	6.7		14.0	77.0	9.1	
University	58.2 (7.6)		23.9	72.3	3.8		17.9	76.8	5.4	
Smoking status (%)		<0.0001				<0.0001				<0.0001
Never smoker	57.8 (7.8)		26.6	70.1	3.3		19.1	75.9	5.1	
Former smoker	59.3 (8.0)		19.9	74.5	5.6		14.4	77.8	7.7	
Current smoker	60.8 (8.3)		15.6	75.9	8.6		11.5	78.1	10.4	
Alcohol intake (g/day) ^2^			10.9 (12.9)	13.0 (14.1)	14.5 (15.8)	<0.0001	4.5 (6.7)	5.3 (7.4)	6.6 (9.4)	<0.0001
Physical activity (%)		<0.0001				0.026				<0.0001
Low	59.3 (8.2)		21.7	72.3	6.0		15.7	76.7	7.7	
Moderate	58.7 (7.9)		20.9	74.2	4.9		16.2	77.7	6.2	
High	58.4 (8.0)		23.4	72.0	4.6		17.6	75.9	6.5	
Occupational status (%)		<0.0001				<0.0001				<0.0001
Unemployed	60.9 (8.6)		14.0	76.3	9.7		12.2	76.5	11.3	
Student	60.9 (8.6)		10.7	78.7	10.7		13.4	74.9	11.7	
Self-employed, farmer	59.4 (8.0)		21.0	73.1	5.9		13.0	79.5	7.5	
Employee, manual worker	59.7 (8.0)		17.1	75.5	7.4		14.7	77.7	7.6	
Managerial staff, intellectual profession	57.3 (7.5)		26.9	70.5	2.6		20.2	75.8	4.1	
Intermediate professions	58.3 (7.7)		21.0	73.2	5.7		17.8	77.0	5.3	
Retired	58.7 (8.0)		21.7	73.2	5.1		16.2	77.3	6.5	
Monthly income per household unit (%) ^3^		<0.0001				<0.0001				<0.0001
Less than 900 €	61.4 (8.8)		16.6	73.0	10.5		11.0	75.8	13.2	
Between 900 € and 1200 €	59.7 (8.1)		16.8	75.8	7.4		15.0	76.6	8.4	
Between 1200 € and 1800 €	59.4 (8.1)		20.4	73.2	6.4		14.2	78.3	7.5	
Between 1800 € and 2300 €	58.9 (7.8)		20.4	74.1	5.5		16.3	77.4	6.2	
Between 2300 € and 2700 €	58.3 (7.8)		21.0	74.7	4.3		18.1	76.5	5.5	
Between 2700 € and 3700 €	57.7 (7.6)		24.4	71.9	3.8		19.0	76.4	4.6	
More than 3700 €	57.3 (7.6)		26.2	70.4	3.4		20.2	75.7	4.1	
Unwilling to answer	58.5 (8.0)		22.6	73.6	3.8		17.7	75.9	6.4	
Matrimonial status (%)		<0.0001				<0.0001				<0.0001
Living alone	60.2 (8.4)		18.4	73.5	8.1		13.0	77.2	9.8	
Living with a partner	59.3 (7.9)		19.1	73.9	7.1		14.5	78.3	7.2	
Married	57.9 (7.8)		24.0	71.9	4.1		19.0	75.8	5.2	
Divorced, separated, widowed	59.7 (8.2)		19.1	73.9	7.1		14.4	77.7	7.9	
BMI (kg/m^2^) ^2^			24.8 (3.6)	25.2 (3.8)	26.3 (4.6)	<0.0001	23.4 (4.5)	23.7 (4.6)	24.3 (5.3)	<0.0001
Nutritional status (%)		<0.0001 ^4^				<0.0001 ^4^				<0.0001 ^4^
Underweight (<18.5 kg/m^2^)	58.8 (8.4)		21.3	69.7	9.0		18.7	74.3	7.0	
Normal (18.5–24.99 kg/m^2^)	58.5 (7.9)		23.5	72.6	3.9		16.9	77.0	6.0	
Overweight (25–29.99 kg/m^2^)	58.9 (8.1)		21.4	72.9	5.7		15.4	77.3	7.3	
Obese (≥30 kg/m^2^)	60.0 (8.5)		17.1	74.4	8.5		14.4	76.2	9.4	
Obese Class I (30–34.99 kg/m^2^)	59.8 (8.3)		17.1	74.8	8.1		14.2	76.9	8.9	
Obese Class II (35–39.99 kg/m^2^)	60.3 (8.6)		17.7	75.3	7.0		15.1	75.0	9.9	
Obese Class III (≥40 kg/m^2^)	60.8 (9.1)		15.1	67.1	17.8		13.9	74.4	11.7	

Boldface indicates statistical significance; ^1^ Adjusted *p* values for multiple comparisons (Hochberg method) based on the Student’s *t* test and Pearson χ^2^ test, as appropriate; ^2^ Mean (SD); ^3^ The household income per month was calculated by consumption units (CU). The number of people of the household was converted into a number of CU according to a weighting system: one CU is attributed for the first adult in the household, 0.5 for other persons aged 14 or older and 0.3 for children under 14 [34]; ^4^ The tests were performed for underweight, normal, overweight and global obesity categories.

**Table 3:** Multinomial logistic regression analyses showing the association between BIS-11 levels and BMI categories (with normal weight category as reference), according to gender in 51,043 participants of the NutriNet-Santé study (2014).

	Over-Control (<52)		Average Impulsivity (52–71)	High Impulsivity (>71)	
	OR (95% CI)	*p* ^1^		OR (95% CI)	*p* ^1^
Men (*n* = 11,929)					
Raw model ^2^					
Underweight	0.94 (0.61, 1.47)	0.80	Reference	2.44 (1.29, 4.63)	0.07
Overweight	0.91 (0.83, 1.00)	0.30	Reference	1.47 (1.23, 1.77)	0.0006
Obese	0.86 (0.78, 0.95)	0.0008	Reference	1.58 (1.40, 1.79)	<0.0001
Obese Class I	0.71 (0.59, 0.85)	0.003	Reference	2.05 (1.56, 2.69)	<0.0001
Obese Class II	0.73 (0.50, 1.07)	0.52	Reference	1.75 (0.98, 3.14)	0.36
Obese Class III	0.69 (0.36, 1.34)	0.70	Reference	5.00 (2.68, 9.34)	<0.0001
Adjusted model ^2,3^					
Underweight	1.05 (0.67, 1.66)	0.90	Reference	1.80 (0.92, 3.51)	0.90
Overweight	0.97 (0.87, 1.06)	0.90	Reference	1.40 (1.16, 1.70)	0.01
Obese	0.80 (0.67, 0.95)	0.16	Reference	1.80 (1.39, 2.33)	0.0002
Obese Class I	0.79 (0.66, 0.96)	0.25	Reference	1.74 (1.31, 2.31)	0.0028
Obese Class II	0.82 (0.55, 1.21)	0.90	Reference	1.42 (0.78, 2.58)	0.90
Obese Class III	0.81 (0.42, 1.57)	0.90	Reference	3.57 (1.86, 6.85)	0.0028
Women (*n* = 39,144)					
Raw model ^2^					
Underweight	1.15 (1.03, 1.28)	0.13	Reference	1.21 (1.02, 1.43)	0.20
Overweight	0.91 (0.85, 0.98)	0.08	Reference	1.21 (1.09, 1.34)	0.003
Obese	0.71 (0.60, 0.84)	0.031	Reference	2.17 (1.70, 2.77)	<0.0001
Obese Class I	0.84 (0.75, 0.95)	0.052	Reference	1.48 (1.28, 1.72)	<0.0001
Obese Class II	0.91 (0.75, 1.11)	0.70	Reference	1.69 (1.34, 2.13)	0.0002
Obese Class III	0.85 (0.63, 1.15)	0.70	Reference	2.00 (1.44, 2.79)	0.0006
Adjusted model ^2,3^					
Underweight	1.17 (1.05, 1.31)	0.092	Reference	1.12 (0.95, 1.33)	0.90
Overweight	0.95 (0.88, 1.02)	0.90	Reference	1.13 (1.02, 1.25)	0.31
Obese	0.95 (0.86, 1.05)	0.90	Reference	1.30 (1.15, 1.48)	0.0013
Obese Class I	0.92 (0.81, 1.04)	0.90	Reference	1.26 (1.08, 1.47)	0.058
Obese Class II	1.04 (0.85, 1.26)	0.90	Reference	1.34 (1.05, 1.70)	0.25
Obese Class III	0.98 (0.72, 1.33)	0.90	Reference	1.48 (1.05, 2.08)	0.90

Boldface indicates statistical significance; ^1^ Adjusted *p* values for multiple comparisons (Hochberg method); ^2^ BMI categories are defined as follow: underweight (<18.5 kg/m^2^), overweight (25–29.99 kg/m^2^), Obese (≥30 kg/m^2^), (obese Class I (30–34.99 kg/m^2^), obese Class II (35–39.99 kg/m^2^), and obese Class III (≥40 kg/m^2^); ^3^ Adjusted for age, education level, smoking status, daily alcohol intake, physical activity, employment status, monthly household income, and matrimonial status.
